# Antioxidant Phenolic Compounds of Cassava (*Manihot esculenta*) from Hainan

**DOI:** 10.3390/molecules161210157

**Published:** 2011-12-07

**Authors:** Bo Yi, Lifei Hu, Wenli Mei, Kaibing Zhou, Hui Wang, Ying Luo, Xiaoyi Wei, Haofu Dai

**Affiliations:** 1 Key Laboratory of Plant Resources Conservation and Sustainable Utilization, South China Botanical Garden, Chinese Academy of Sciences, Xingke Road 723, Tianhe District, Guangzhou 510650, China; 2 Hainan Key Laboratory for Research and Development of Natural Product from Li Folk Medicine, Institute of Tropical Bioscience and Biotechnology, Chinese Academy of Tropical Agricultural Sciences, Haikou 571101, China; 3 Graduate School of Chinese Academy of Sciences, Yuquanlu 19A, Beijing 100049, China; 4 Pharmacy Department of 187 Hospital PLA, Longkun South Road 100, Haikou 571159, China

**Keywords:** *Manihot esculenta*, isolation, phenolic compounds, DPPH, ABTS, antioxidant activity

## Abstract

An activity-directed fractionation and purification process was used to isolate antioxidant components from cassava stems produced in Hainan. The ethyl acetate and *n*-butanol fractions showed greater DPPH˙and ABTS·^+^ scavenging activities than other fractions. The ethyl acetate fraction was subjected to column chromatography, to yield ten phenolic compounds: Coniferaldehyde (**1**), isovanillin (**2**), 6-deoxyjacareubin (**3**), scopoletin (**4**), syringaldehyde (**5**), pinoresinol (**6**), *p*-coumaric acid (**7**), ficusol (**8**), balanophonin (**9**) and ethamivan (**10**), which possess significant antioxidant activities. The relative order of DPPH· scavenging capacity for these compounds was ascorbic acid (reference) > **6** > **1** > **8** > **10** > **9** > **3** > **4** > **7** > **5** > **2**, and that of ABTS·^+^ scavenging capacity was **5** > **7** > **1** > **10** > **4** > **6** > **8** > **2** > Trolox (reference compound) > **3** > **9**. The results showed that these phenolic compounds contributed to the antioxidant activity of cassava.

## 1. Introduction

Cassava (*Manihot esculenta* Crantz) is one of the most important food crops in the tropical regions of the World, being a staple source of energy carbohydrates for human consumption. Isolated cassava starch is a functional ingredient used in food, paper, textile and pharmaceutical industries and has economic value for the starch exporting countries [1]. The high starch production attributes, together with the unique properties of its starch make cassava suitable for particular food and non-food applications [2,3]. Cassava roots easily deteriorate during storage soon after harvest. When cassava root is damaged by cutting or fungal infection, the tissues accumulate phenolic compounds, such as scopolin, scopoletin, and diterpenoid compounds in the injured or infected regions [4,5]. In China, cassava is mostly found distributed in Hainan, Guangdong, Guangxi, Guizhou, Yunnan, Fujian, and Taiwan provinces*.* Currently, cassava stems is still under-utilized in China and no phytochemical investigations on them have been carried out. In this context, it is desirable to seek higher value products from cassava stems which could be economically worthwhile for commercial exploitation.

In recent years, there has been increasing evidence to suggest that many age-related human diseases such as heart disease, cancer, arthritis, immune system decline, brain dysfunction, and cataracts are the results of cellular damage by free radicals, and that antioxidants in our diet could play an important role in the prevention of such diseases [6,7]. This has fueled much public interest in natural antioxidants and has led to an extensive search for effective antioxidants in Nature [8,9], especially those that are present naturally in human diets [10,11]. The ability of phenolic compounds to serve as antioxidants has been recognized, leading to speculation about the potential benefits of ingesting phenolic-rich foods [12,13]. The aim of this research was to examine the antioxidant properties of fractions and phenolic compounds derived from the ethanol extract of the stem of cassava for use as natural antioxidants.

## 2. Results and Discussion

### 2.1. Isolation of Antioxidant Compounds

**Figure 1:**
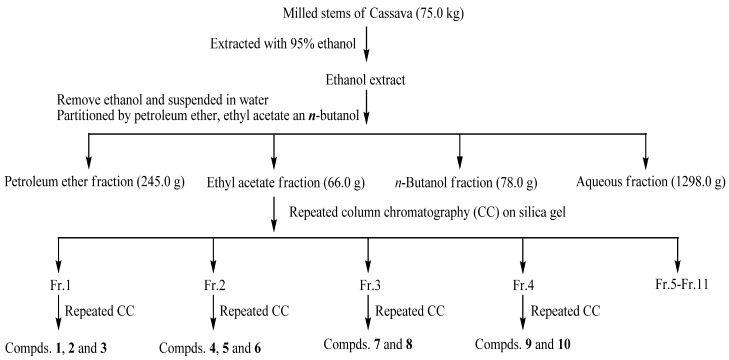
Extraction, fractionation and column chromatography separation of cassava stems.

The stems of cassava were milled and extracted with 95% ethanol at room temperature. After evaporation, the residue was suspended in H_2_O and successively partitioned with petroleum ether, ethyl acetate and *n*-butanol to obtain the PEF, EAF, BUF and AF fractions, respectively, which were all evaluated for their DPPH˙ and ABTS free radical scavenging ability using a conventional spectrophotometric assay. Among the four fractions, the results suggested that the antioxidants were mainly contained in EAF and BUF fractions. In this paper, further experiments were carried out on the EAF fraction to separate its antioxidant components. The EAF fraction was submitted to successive chromatographic fractionation and purification to yield compounds **1–10**, as shown in Figure 1.

### 2.2. Structural Elucidation of Isolated Compounds

**Figure 2:**
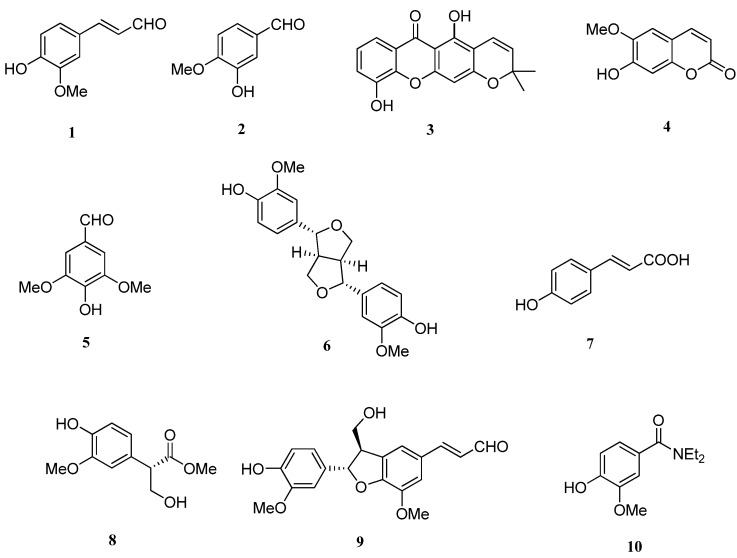
Structures of compounds **1–10**.

The isolated compounds were identified on the basis of spectroscopic analyses, including ^1^H- and ^13^C-NMR (DEPT) spectroscopy, combined with comparison of its NMR data to those reported in the literature. The chemical structures of these isolates were identified as shown in Figure 2.

Isovanillin (**2**), syringaldehyde (**5**), ficusol (**8**) and ethamivan (**10**) are simple phenols, coniferaldehyde (**1**) and *p*-coumaric acid (**7**) are phenylpropanoids, scopoletin (**4**) is a coumarin, 6-deoxyjacareubin (**3**) is a xanthone, and pinoresinol (**6**) and balanophonin (**9**) are lignans. All the compounds except scopoletin (**4**) were isolated from stems of cassava for the first time [14].

### 2.3. DPPH Scavenging Capacities

It is well known that the antioxidant activity of plant extracts containing phenolic compounds is due to their capacity to act as donors of hydrogen atoms or electrons and to capture the free radicals. The DPPH assay is one of the tests commonly used to prove the antioxidant ability of fractions and isolated pure compounds to act as donors of hydrogen atoms [15]. The obtained results in our case are shown in Table 1. Among the fractions, the EAF and BUF fractions were found to be the most potent DPPH radical scavengers, with EC_50_ values of 0.518 and 0.616 mg/mL, respectively, while the PEF was the least active scavenger. The ten phenolic compounds isolated from the DPPH˙ active EAF fraction as well as the positive control, ascorbic acid, were also evaluated and compared for their DPPH˙ scavenging capacities. The relative order of DPPH˙ scavenging capacity for the isolated phenolic compounds was found to be: pinoresinol (**6**) > coniferaldehyde (**1**) > ficusol (**8**) > ethamivan (**10**) > balanophonin (**9**) > 6-deoxyjacareubin (**3**) > scopoletin (**4**) > *p*-coumaric acid (**7**) >syringaldehyde (**5**) > isovanillin (**2**). All phenolic compounds demonstrated lower DPPH˙ scavenging capacities than the positive control, ascorbic acid.

### 2.4. ABTS Free Radical Scavenging Assay

The antioxidant activities of the extracts and the isolated phenolic compounds determined using the ABTS free radical scavenging assay are shown in Table 1. Among the fractions, the EAF fraction was found to be the most potent ABTS free radical scavenger, with an EC_50_ value of 0.257 mg/mL. All the phenolic compounds examined were found to possess good ABTS free radical scavenging activity.

**Table 1:** The DPPH˙ and ABTS·^+^ scavenging activities of the extracts and the isolated phenoliccompounds *and* amounts of total phenolics in fractions ofcassava ^a^.

Test materials	Antioxidant activity		Total phenolics (GAE g/g extract)
	DPPH˙	ABTS·^+^	
PEF	4.394 ± 0.23	3.353 ± 0.25	4.58 ± 1.35 ^b^
EAF	0.518 ± 0.20	0.257 ± 0.07	29.82 ± 1.02 ^a^
BUF	0.616 ± 0.24	0.345 ± 0.08	15.06 ± 0.26 ^b^
AF	2.667 ± 0.11	2.408 ± 0.22	6.02 ± 0.18 ^b^
Conniferaldehyde (**1**)	0.225 ± 0.03	0.046 ± 0.03	
Isovanilin (**2**)	4.654 ± 0.22	0.117 ± 0.05	
6-Deoxyjacareubin (**3**)	1.058 ± 0.15	0.121 ± 0.05	
Scopoletin (**4**)	1.179 ± 0.16	0.053 ± 0.04	
Syringaldehyde (**5**)	1.572 ± 0.21	0.028 ± 0.02	
Pinoresinol (**6**)	0.200 ± 0.01	0.055 ± 0.05	
*p*-Coumaric acid (**7**)	1.327 ± 0.13	0.029 ± 0.03	
Ficusol (**8**)	0.259 ± 0.02	0.058 ± 0.04	
Balanophonin (**9**)	0.716 ± 0.05	0.199 ± 0.17	
Ethamivan (**10**)	0.374 ± 0.03	0.050 ± 0.04	
Ascorbic acid	0.100 ± 0.01	-	
Trolox	-	0.118 ± 0.01	

^a^ PEF, EAF, BUF, and AF represent the petroleum ether fraction, ethyl acetate fraction, *n*-butanol and aqueous fraction of cassava, respectively. The DPPH˙ and ABTS·^+ ^ scavenging activities are represented by their EC_50_ values, and the EC_50_ values of test materials are expressed as mg/mL. Ascorbic acid was used as a positive control for DPPH˙ scavenging activities, and Trolox was used as a positive control for ABTS·^+^ scavenging activities. Values are means ± SD of three determinations. Different letters in the same column indicate significant difference (P < 0.05). Purity (%) of tested compounds were >95%.

The relative order of ABTS scavenging capacity for the isolated phenolic compounds was found to be: Syringaldehyde (**5**) > *p*-coumaric acid (**7**) > coniferaldehyde (**1**) > ethamivan (**10**) > scopoletin (**4**) > pinoresinol (**6**) > ficusol (**8**) > isovanillin (**2**) > 6-deoxyjacareubin (**3**) > balanophonin (**9**). Among the ten compounds, eight demonstrated higher antioxidant activity than the positive control, Trolox. Given the complexity of the structures of these compounds, it is difficult to discuss their structure-activity relationships.

### 2.5. Determination of the Amount of Total Phenolics

Many studies have conclusively revealed that the overall antioxidant activity of plants is largely due to the amount of total phenolics and total flavonoids present [16,17]. Generally, the antioxidant activities of phenolic compounds are realized by inactivating lipid free radicals and preventing decomposition of hydroperoxides into free radicals. The amounts of the total phenolics in the fractions of cassava stems are shown in Table 1. EAF showed the highest amount of total phenolics, followed by BUF, AF, and PEF. At the same time, we could find that this order was similar to that of their antioxidant and radical-scavenging activities. Results in our study also demonstrated that the extent of antioxidant activity of cassava stems was in accordance with the amounts of phenolics present in this species.

## 3. Experimental

### 3.1. General Procedures and Reagents

^1^H- and ^13^C-NMR spectra were obtained on a Bruker AV-400 instrument using deuterated dimethyl sulfoxide (DMSO-*d*_6_), chloroform (CDCl_3_), acetone (CD_3_COCD_3_) and methanol (CD_3_OD) as solvents. Column chromatography was carried out on silica gel (200–300 mesh, Qingdao Marine Chemistry Company, Qingdao, China) and Sephadex LH-20 (Merck, Darmstadt, Germany). Optical density measurements were made with a Shimadzu UV-2550 spectrophotometer (Shimadzu, Kyoto, JP). 1,1-Diphenyl-2-picrylhydrazyl radical (DPPH·), 2,2′-azinobis-(3-ethylbenzothiazoline-6-sulfonate) (ABTS·^+^), 6-hydroxy-2,5,7,8-tetramethylchroman-2-carboxylic acid (Trolox) and ascorbic acid were purchased from Sigma–Aldrich (St. Louis, MO, USA). All other chemicals were of analytical reagent grade and used without any further purification.

### 3.2. Plant Materials

Cassava stems were collected in the experimental field plot of the Institute of Tropical Bioscience and Biotechnology from Wenchang, Hainan, China, in November 2008, and authenticated by Prof. Zhunian Wang, Tropical Crops Genetic Resource Institute, Chinese Academy of Tropical Agriculture Sciences. A voucher specimen of the collection (No. ME-081112) has been deposited at the Institute of Tropical Bioscience and Biotechnology, Chinese Academy of Tropical Agriculture Sciences.

### 3.3. Extraction and Isolation of Antioxidant Compounds

Antioxidants of cassava stems were extracted and fractionated according to their polarity as shown in Figure 1. The fresh, milled stems of cassava (75.0 kg) were exhaustively extracted with 95% ethanol (3 × 20 L, total amount 60 L) three times at room temperature for three weeks. The ethanol extract was then filtered through absorbent gauze, and the filtrate was concentrated under reduced pressure to remove ethanol at 50 °C. The extract (1.8 kg) was suspended in H_2_O (2 L) and partitioned with petroleum ether (1 L × 3). The resulting supernatants were collected and filtered through absorbent gauze, followed by evaporation of the solvent at 50 °C under reduced pressure. The resulting liquid residue was labeled as the petroleum ether fraction. The defatted material remaining from petroleum ether extraction was successively partitioned with ethyl acetate (1 L × 3) and *n*-butanol (1 L × 3). The ethyl acetate and *n*-butanol extracts were separately combined and evaporated to dryness under reduced pressure, while the aqueous layer was lyophilised to dryness. These four fractions were designated as PEF (245.0 g), EAF (66.0 g), BUF (78.0 g) and AF (1,298.0 g), respectively. The EAF fraction (66.0), which showed potent antioxitant activity, was subjected to silica gel CC (100 × 40 cm, 3.5 kg) eluted with increasing polarities of a mixture of chloroform and methanol (100:1, 80:1, 50:1, 30:1, 15:1, 10:1, 5:1, 2:1, 1:1, 0:1, each 4.0 L) to yield 11 fractions (Fr. 1–Fr. 11). Repeated CC on silica gel CC (2.0 × 45 cm, 25 g) eluted with petroleum ether–acetone gradients (10:1–2:1, v/v,) and Sephadex LH-20 (3 × 100 cm, CHCl_3_–MeOH, 1:1, v/v) led to the isolation of compounds **1** (16.7 mg), **2** (43.2 mg) and **3** (13.9 mg) from Fr. 1 (6.2 g). Fr. 2 (4.0 g) yielded compounds **4** (850.3 mg), **5** (5.8 mg) and **6** (15.3 mg) after CC with Sephadex LH-20 (3 × 100 cm, CHCl_3_–MeOH, 1:1, v/v, 3.0 L) and further purification with silica gel CC (2.0 × 45 cm, 25 g, CHCl_3_–MeOH, 30:1). Compounds **7** (5.3 mg) and **8** (28.4 mg) were obtained from Fr. 3 (5.2 g) by repeated CC on Sephadex LH-20 (CHCl_3_-MeOH, 1:1, v/v, 2.0 L) and silica gel CC (CHCl_3_-MeOH, 15:1, v/v). From Fr. 4 (16.0 g), compounds **9** (49.6 mg) and **10** (9.1 mg) were separated by silica gel CC (CHCl_3_-MeOH, 9:1) and Sephadex LH-20 (3 × 100 cm) eluting with EtOH. The fractions 5 to 11 which possess a similar antioxidant activity are being isolated and studied.

### 3.4. Spectroscopic Data

*Coniferaldehyde* (**1**): C_10_H_10_O_3_, colorless needles, ^1^H-NMR (CDCl_3_), *δ* 7.63 (1H, d, *J* = 7.6 Hz, H-9), 7.39 (1H, d, *J *= 15.8 Hz, H-7), 7.11 (1H, dd, *J* = 8.2, 1.8 Hz, H-5),7.06 (1H, d, *J* = 1.8, H-3), 6.95 (1H, d, *J* = 8.2 Hz, H-6), 6.58 (1H, dd, *J* = 15.8, 7.6 Hz, H-8), 3.93 (3H, s, OCH3); ^13^C-NMR (CDCl_3_), *δ* 149.0 (s, C-1), 147.0 (s, C-2), 109.6 (d, C-3), 126.6 (s, C-4), 124.0 (d, C-5), 114.9 (d, C-6), 153.0 (d, C-7), 126.5 (d, C-8), 193.6 (d, C-9), 56.0 (OCH_3_). The above data were identical to those in the literature [18].

*Isovanillin* (**2**): C_8_H_8_O_3_, crystals, ^1^H-NMR (CDCl_3_), *δ* 9.83 (1H, s, CHO), 7.42 (1H, dd, *J* = 8.4, 1.7 Hz, H-6), 7.42 (1H, d, *J* = 1.7 Hz, H-2), 7.04 (1H, d, *J* = 8.4 Hz, H-5), 3.97 (3H, s, OCH_3_); ^13^C-NMR (CDCl_3_), *δ* 130.0 (s, C-1), 108.8 (d, C-2), 147.2 (s, C-3), 151.7 (s, C-4), 114.4 (d, C-5), 127.5 (d, C-6), 190.8 (CHO), 56.1 (OCH_3_). The above data were consistent with the literature data [19].

6-*Deoxyjacareubin* (**3**): C_18_H_14_O_5_, yellow needles, ^1^H-NMR (acetone-*d_6_*), *δ* 13.33 (1H, s, OH-1), 7.66 (1H, dd, *J* = 7.8, 1.6 Hz, H-8), 7.35 (1H, dd, *J* = 7.8, 1.6 Hz, H-6), 7.27 (1H, t, *J* = 7.8 Hz, H-7), 6.69 (1H, d, *J* = 10.1 Hz, H-1′), 6.40 (1H, s, H-4), 5.76 (1H, d, *J* = 10.1 Hz, H-2′), 1.48 (6H, s, H-4′,5′); ^13^C- NMR (acetone-*d*_6_), *δ* 162.7 (s, C-1), 106.3 (s, C-2), 159.5 (s, C-3), 96.7 (d, C-4), 148.0 (s, C-5), 122.6 (d, C-6), 126.0 (d, C-7), 116.7 (d, C-8), 182.9 (s, C-9), 117.2 (d, C-1′), 129.9 (d, C-2′), 80.2 (s, C-3′), 29.6 (q, C-4′), 29.6 (q, C-5′), 159.5 (s, C-4a), 123.1 (s, C-8a), 105.2 (s, C-9a), 147.0 (s, C-10a). Its NMR data were accord with the reported data [20].

*Scopoletin* (**4**): C_10_H_8_O_4_, yellow needles, ^1^H-NMR (DMSO-*d_6_*), *δ* 7.89 (1H, d, *J* = 9.0 Hz, H-4), 7.20 (1H, s, *J* = 7.20 Hz, H-5), 6.78 (1H, s, H-8), 6.21 (1H, d, *J* = 9.0, H-3), 3.81 (3H, s, OCH3); ^13^C-NMR (DMSO-*d_6_*), *δ* 160.6 (s, C-2), 111.6 (d, C-3), 144.3 (d, C-4), 109.5 (d, C-5), 145.1 (s, C-6), 151.0 (s, C-7), 102.7 (d, C-8), 149.4 (s, C-9), 110.5 (s, C-10), 55.9 (OCH_3_). The above data were identical to the literature data [21].

*Syringaldehyde* (**5**): C_9_H_10_O_4_, yellow amorphous powder, ^1^H-NMR (CDCl_3_), *δ* 9.82 (1H, s, H-7), 7.15 (2H, s, H-2, 6), 3.97 (6H, s, 2×OCH_3_); ^13^C-NMR (CDCl_3_), *δ* 128.6 (s, C-1), 140.9 (s, C-2, 4), 147.4 (s, C-3, 5), 106.8 (d, C-2, 6), 190.7(d, C-7), 56.9 (OCH_3_). The above data were identical to the literature data [22].

Pinoresinol (**6**): C_20_H_22_O_6_, colorless crystals, ^1^H-NMR (CDCl_3_), *δ* 6.90 (2H, brs, H-2, 2′), 6.88 (2H, d, *J* = 8.0 Hz, H-5, 5′), 6.81 (2H, dd, *J* = 8.0, 1.9 Hz, H-6, 6′), 4.47 (2H, d, *J* = 4.4 Hz, H-7, 7′), 4.24 (2H, m, H-9b, 9′b), 3.90 (6H, s, 2×OCH_3_), 3.88 (2H, m, H-9a, 9′a), 3.10 (2H, m, H-8, 8′); ^13^C-NMR (CDCl_3_), *δ* 132.8 (s, C-1, 1′), 108.6 (d, C-2, 2′), 145.2 (s, C-3, 3′), 146.7 (s, C-4, 4′), 114.3 (d, C-5, 5′), 119.0 (d, C-6, 6′), 85.8 (d, C-7, 7′), 54.1 (d, C-8, 8′), 71.6 (t, C-9, 9′), 56.0 (OCH_3_). The above data were identical to the literature data [18].

*p*-*Coumaric acid* (**7**): C_9_H_8_O_3_, colorless needles, ^1^H-NMR (CD_3_OD), *δ* 7.56 (1H, d, *J* = 15.8, H-7), 7.42 (2H, d, *J* = 8.6 Hz, H-2, 6), 6.77 (2H, d, *J* = 8.6 Hz, H-3, 5), 6.25 (1H, d, *J* = 15.8 Hz, H-8); ^13^C- NMR (CD_3_OD), *δ* 171.2 (s, C-9), 161.1 (s, C-4), 146.6 (d, C-7), 131.1 (s, C-2,6), 127.3 (s, C-1), 116.9 (d, C-3, 5), 115.8 (d, C-8). The above data were identical to the literature data [23].

*Ficusol* (**8**): C_11_H_14_O_5_, oil, ^1^H-NMR (CDCl_3_), *δ* 6.86 (1H, d, *J* = 8.0, H-5′), 6.77 (1H, d, *J* = 1.8 Hz, H-2′), 6.74 (1H, dd, *J* = 8.0, 1.8 Hz, H-6′), 4.11 (1H, m, H-3a), 3.79 (1H, m, H-3b), 3.75 (1H, m, H-2); ^13^C-NMR (CDCl_3_), *δ* 173.8 (s, C-1), 53.5 (d, C-2), 64.6 (t, C-3), 127.3 (s, C-1′), 114.7 (d, C-2′), 145.3 (s, C-3′), 146.7 (s, C-4′), 110.6 (d, C-5′), 121.1 (d, C-6′), 52.2 (1-OCH_3_), 56.0 (3′-OCH_3_). The above data were identical to the literature data [15].

*Balanophonin* (**9**): C_20_H_20_O_6_, colorless crystal, ^1^H-NMR (CDCl_3_), *δ* 9.61 (1H, d, *J* = 7.7 Hz, H-9′), 7.40 (1H, d, *J* = 15.8 Hz, H-7′), 7.13 (1H, s, H-2′), 7.03 (1H, s, H-6′), 6.88 (1H, d, *J* = 7.4 Hz, H-6), 6.87 (1H, d, *J* = 1.8 Hz, H-2), 6.59 (1H, dd, *J* = 15.8, 7.7 Hz, H-8′), 5.62 (1H, d, *J* = 7.0 Hz, H-7), 4.88 (1H, d, *J* = 7.4 Hz, H-5), 3.94 (2H, d, *J* = 6.2 Hz, H-9), 3.92 (1H, s, OCH_3_), 3.86 (1H, s, OCH_3_), 3.65 (1H, td, *J* = 6.0 Hz, H-8); ^13^C-NMR (CDCl_3_), *δ* 129.3 (s, C-1), 108.8 (d, C-2), 146.8 (s, C-3), 145.9 (s, C-4), 144.5 (d, C-5), 119.4 (d, C-6), 89.0 (d, C-7), 53.0 (d, C-8), 63.9 (t, C-9), 56.0 (OCH3). 128.0 (s, C-1′), 118.2 (d, C-2′), 144.7 (s, 3′), 151.5 (s, C-4′), 132.3 (d, C-5′), 112.3 (d, C-6′), 153.1 (d, C-7′), 126.3 (d, C-8′), 193.6 (CHO, C-9′), 56.1 (3-OCH_3_), 56.1 (3′-OCH_3_). The above data were identical to the literature data [24].

*Ethamivan* (**10**): C_12_H_17_NO_3_, colorless needles, ^1^H-NMR (CD_3_OD), *δ* 7.57 (1H, d, *J* = 1.8, H-2), 7.48 (1H, dd, *J* = 8.2, 1.8 Hz, H-6), 6.77 (1H, d, *J* = 8.2 Hz, H-5), 3.88 (3H, s, OCH_3_), 3.02 (4H, q, *J* = 7.3 Hz, H-8,8′), 1.29 (6H, t, *J* = 7.3 Hz, H-9,9′); ^13^C-NMR (CD_3_OD), *δ* 129.7 (s, C-1), 115.3 (d, C-2), 148.2 (s, C-3), 150.3 (s, C-4), 114.0 (d, C-5), 124.3 (d, C-6), 175.0 (s, C-7), 56.4 (q, OCH_3_), 43.4 (t, C-8,8′), 11.6 (q, C-9,9′). The above data were identical to the literature data [25].

### 3.5. DPPH˙ Scavenging Capacity

The DPPH˙ scavenging capacity was measured using the method of Zhang *et al.* [9] and Lu and Yeap Foo [26] with modifications. A 0.1 mM solution of DPPH˙ in ethanol was prepared and this solution (2 mL) was added to antioxidant solutions in DMSO of different concentrations (0.1 mL). After gentle mixing and 30 min of standing at room temperature, the absorbance of the resulting solutions was measured at 517 nm. Ascorbic acid was used as a positive control. The percentage scavenging effect was calculated as:

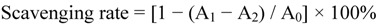

where A_0_ was the absorbance of the control (without extract), A_1_ was the absorbance in the presence of the extract, A_2_ was the absorbance without DPPH˙. The EC_50_ value, defined as the amount of antioxidant necessary to decrease the initial DPPH˙ concentration by 50%, was calculated from the results.

### 3.6. ABTS Free Radical Scavenging Assay

The ABTS free radical scavenging ability was carried by a modified method as described by Fang *et al.* [27] and Re *et al.* [28]. Potassium persulfate was added to 7 mM of ABTS·^+^ and kept for 12–16 h at room temperature in the dark. The ABTS·^+^ solution was diluted with PBS (potassium phosphate-buffered saline, pH 7.4) to an absorbance of 0.70 ± 0.02 at 734 nm before analysis. ABTS·^+^ cation solution (3.0 mL) was added to antioxidant solutions in DMSO of different concentrations (0.1 mL) and mixed thoroughly. The reaction mixture was kept at room temperature for 6 min, and the absorbance was recorded at 734 nm on the Shimadzu UV-2550 spectrophotometer. Trolox was used as a positive control. The percentage scavenging effect was calculated as:

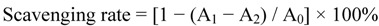

where A_0_ was the absorbance of the control (without extract), A_1_ was the absorbance in the presence of the extract, A_2_ was the absorbance without ABTS·^+^. The EC_50_ value, defined as the amount of antioxidant necessary to decrease the initial ABTS·^+^ concentration by 50%, was calculated from the results.

### 3.7. Determination of the Amount of Total Phenolics

The amount of total phenolic contents was determined according to the Folin-Ciocalteu method [29] with some modification. Briefly, sample fractions (1.0 mL) were mixed with distilled water (9.0 mL) in a 25 mL volumetric flask. Then Folin–Ciocalteu’s phenol reagent (1.0 mL) was added to the mixture and then shaken. The mixture was kept within 5 min, followed by the addition of 7% Na_2_CO_3_ solution (10 mL). The mixed solution was then diluted to 25 mL with distilled water and mixed thoroughly. After 90 min of reaction at room temperature, the absorbance *versus* a blank was measured at 750 nm. The standard curve for total phenolics was developed using gallic acid standard solution (0–100 mg/L). The total phenolics in the extract and the fractions were expressed as g gallic acid equivalents (GAE)/g extract. All samples were tested in three times, and the results were averaged.

### 3.8. Statistical Analyses

Data were expressed as means ± standard deviation (S.D.) of three parallel measurements. Statistical calculations were carried out by SAS. Analysis of variance was performed by the ANOVA procedures. Duncan’s new multiple-range test was used to determine the difference of means. Analysis of regression was performed by the REG procedures.

## 4. Conclusions

In the present research, two methods have been used to determine the antioxidant capacity of various extracts and ten isolates from the stems of cassava. All the tested extracts of cassava showed antioxidant and radical-scavenging activities, and the order of antioxidant and radical-scavenging activities among the extracts assayed through all the two methods was found to be EAF > BUF > AF > PEF. This order was similar to the total phenolics contents of the fractions. Ten antioxidant compounds: coniferaldehyde (**1**), isovanillin (**2**), 6-deoxyjacareubin (**3**), scopoletin (**4**), syringaldehyde (**5**), pinoresinol (**6**), *p*-coumaric acid (**7**), ficusol (**8**), balanophonin (**9**) and ethamivan (**10**) were isolated and identified for the first time from stems of cassava by an activity-guided isolation and were found to have DPPH˙ scavenging capacity and ABTS free radical scavenging ability. However, the antioxidant and radical-scavenging activities of some compounds isolated from the stems of cassava were discordant in the two assay methods. Therefore, we should use at least two methods to evaluate whether a sample has antioxidant activity. These phenolic compounds are the main constituents contributing to the antioxidant activities of the ethyl acetate fraction of stems of cassava, and might be used as natural antioxidants and alternatives to synthetic antioxidants.
